# Supplementary material for: Trace Immunosensing of Multiple Neonicotinoid Insecticides by a Novel Broad-Specific Antibody Obtained from a Rational Screening Strategy
Source: Biosensors (Basel). 2022 Sep 3;12(9):716. doi: 10.3390/bios12090716 (PMC9496258; doi:10.3390/bios12090716)
Supplement: Supplementary file 1 [file biosensors-12-00716-s001.zip › biosensors-1876137-supplementary.pdf]

Trace Immunosensing of Multiple Neonicotinoid Insecticides by a Novel Broad-Specific Antibody Obtained from a Rational Screening Strategy

Shasha Jiao [1], Yan Wang [1], Yunyun Chang [1], Pengyan Liu [1], Yang Chen [1], Yihua Liu [2], Guonian Zhu [1] and Yirong Guo [1,*]

[1] Institute of Pesticide and Environmental Toxicology, Key Laboratory of Biology of Crop Pathogens and Insects of Zhejiang Province, Ministry of Agriculture Key Laboratory of Molecular Biology of Crop Pathogens and Insects, Zhejiang University, Hangzhou 310058, China
[2] Research Institute of Subtropical Forestry, Chinese Academy of Forestry, Hangzhou 311400, China
* Correspondence: yirongguo@zju.edu.cn; Tel.: +86-571-88982683

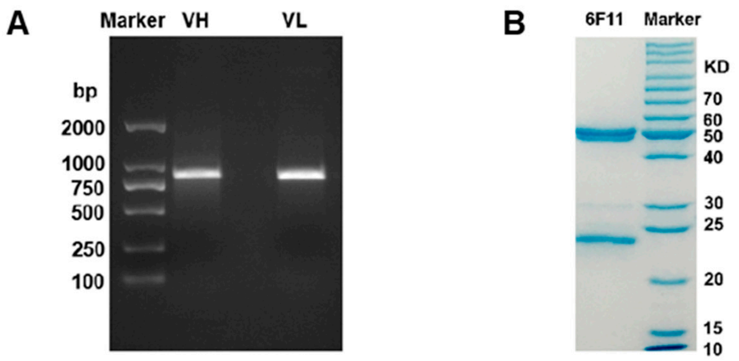

Figure S1. Agarose gel electrophoresisin of the PCR product (**A**). SDS-page of the full-length rAb expressed in mammalian cells (**B**).

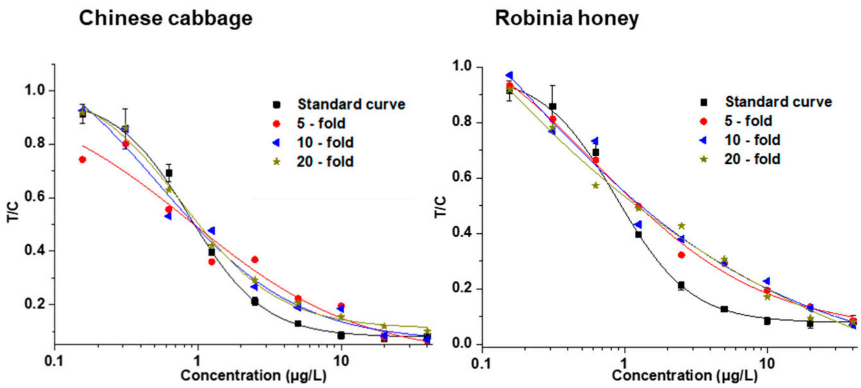

Figure S2. Evaluation of matrix effects in GNISassays of imidacloprid.
